# The Role of Sphingolipids and Specialized Pro-Resolving Mediators in Alzheimer’s Disease

**DOI:** 10.3389/fimmu.2020.620348

**Published:** 2021-01-29

**Authors:** Nienke M. de Wit, Kevin Mol, Sabela Rodríguez-Lorenzo, Helga E. de Vries, Gijs Kooij

**Affiliations:** Department of Molecular Cell Biology and Immunology, Amsterdam Neuroscience, Amsterdam UMC, Vrije Universiteit Amsterdam, Amsterdam, Netherlands

**Keywords:** Alzheimer’s disease, neuroinflammation, sphingolipids, specialized pro-resolving mediator, sphingosine-1-phosphate, ceramide, bioactive lipids

## Abstract

Alzheimer’s disease (AD) is the leading cause of dementia worldwide giving rise to devastating forms of cognitive decline, which impacts patients’ lives and that of their proxies. Pathologically, AD is characterized by extracellular amyloid deposition, neurofibrillary tangles and chronic neuroinflammation. To date, there is no cure that prevents progression of AD. In this review, we elaborate on how bioactive lipids, including sphingolipids (SL) and specialized pro-resolving lipid mediators (SPM), affect ongoing neuroinflammatory processes during AD and how we may exploit them for the development of new biomarker panels and/or therapies. In particular, we here describe how SPM and SL metabolism, ranging from ω-3/6 polyunsaturated fatty acids and their metabolites to ceramides and sphingosine-1-phosphate, initiates pro- and anti-inflammatory signaling cascades in the central nervous system (CNS) and what changes occur therein during AD pathology. Finally, we discuss novel therapeutic approaches to resolve chronic neuroinflammation in AD by modulating the SPM and SL pathways.

## Introduction

The central nervous system (CNS) is one of the most important but vulnerable parts of the human body. CNS-specific cell types, for example microglia, oligodendrocytes, and astrocytes, play a vital role in securing CNS homeostasis and supporting neuronal functioning. In addition, the unique properties of the microvasculature of the CNS that forms the blood brain barrier (BBB), further ensures a tightly controlled CNS environment. The BBB consists of endothelial cells, which are supported by pericytes and astrocytes, together regulating the flow of molecules and cells in and out of the CNS to safeguard its homeostasis (1–5). Over the past decades, worldwide occurrences of neurodegenerative diseases, such as Alzheimer’s disease (AD), are increasing and it is expected that this trend will continue (6). Despite years of research and increasing fundamental knowledge, only a few treatments have been developed and used, but none of such interventions results in curing these devastating neurodegenerative diseases, thereby creating a high and unmet clinical need. For this, more fundamental insight into pathological mechanisms that underlie AD pathology is therefore needed to facilitate the development of potential novel treatment regimes.

Key modulators of a variety of physiological (including cellular) processes are lipids. Lipids are highly abundant in the dry mass of the CNS (up to 50%) where they serve important biological functions. Apart from being structural components of a cell membrane, lipids also act as energy storage source and play important roles in cell signaling pathways such as maintaining BBB homeostasis, immune regulation, and myelination (7–9). Since lipid metabolism occurs in such core CNS processes, alterations in lipid metabolism influences the pathophysiology of various neurodegenerative diseases (10). Therefore, targeting lipid metabolism may result in new perspectives for the treatment of such diseases.

Excessive or uncontrolled inflammation is known as a unifying feature of a plethora of chronic diseases, including neurodegenerative diseases like AD (11, 12). It has become clear that lipids and their metabolites can influence the immune responses and inflammatory processes, in promoting as well as in resolving inflammation. In this review we will discuss how bioactive lipids, including sphingolipids (SLs) and specialized pro-resolving mediators (SPMs), are involved in chronic neuroinflammation in AD and how such bioactive lipids can be used for the development of new therapies.

## Alzheimer’s Disease

AD is the predominant cause of dementia with an estimated 54 million cases worldwide, and with an expected growth to 130 million cases by 2050 (13). It is a progressive mental disorder that is characterized by cognitive impairment and memory loss. Next to age, the ϵ4 allele of the apolipoprotein E gene (ApoE) is the strongest genetic risk factor for AD (14). Next to peripheral tissues involved in cholesterol metabolism, ApoE is highly expressed in the brain where it plays an important role in lipid trafficking (15). Moreover, it is involved in synaptic plasticity, synaptogenesis, inflammation, blood-brain barrier function and in regeneration after injury (16, 17). However, how ApoE contributes to AD remains to be elucidated.

The major neuropathological hallmarks of AD are the accumulation of extracellular senile plaques composed of aggregating β-amyloid (Aβ) and the intracellular aggregation of hyperphosphorylated tau protein. Aβ is released as monomer into the extracellular environment when β-amyloid precursor protein APP is processed by the amyloidogenic pathway (18, 19). The monomers can aggregate to form oligomers, protofibrils, fibrils and, ultimately, plaques, all of which can have neurotoxic effects causing synaptic dysfunction, reactive oxygen species (ROS) formation, increased membrane permeability, and disrupted mitochondrial and proteasomal processes (20–25). Tau is a neuronal microtubule-associated protein that is distributed to the axons to regulate microtubule assembly and stability (26–28). However, when tau is hyperphosphorylated, as seen in AD, it becomes sequestered into neurofibrillary tangles (NFTs), which are mainly found in neuronal processes known as neuropil threads or dystrophic neurites. The dissociation of tau proteins from microtubules negatively affects synaptic plasticity, leading to neurodegeneration (29–31).

Another neuropathological process in AD is neuro-inflammation. Neuro-inflammation describes the reactive morphology and altered function of the glial compartment (32). Although the observed inflammatory glial response is presumed to be secondary to neuronal death or dysfunction, it is suggested that the activation of microglia and astrocytes contributes to the progression of AD. The main cellular players in neuroinflammatory processes are microglia, the innate immune cells of the CNS. Microglia have a complex function that involves an anti-inflammatory (pro-resolving) role where they engulf toxic proteins and apoptotic cells or a (chronic) pro-inflammatory phenotype, that promotes neurotoxicity through excessive production and secretion of inflammatory mediators. Chronically activated microglia release pro-inflammatory mediators like interleukin-1β (IL-1β), IL-6, IL-12, tumor necrosis factor-α (TNF-α), ROS, superoxide, and nitric oxide (NO) causing CNS tissue damage (33–36). On the other hand, pro-resolving microglia are involved in the healing phases of CNS injury by actively monitoring and controlling the extracellular environment (37). In addition, by secreting anti-inflammatory mediators like IL-10 and transforming growth factor β (TGF-β), these cells are able to prevent neurotoxicity, thereby restoring CNS homeostasis (38). During homeostatic conditions, the pro-inflammatory response of microglia is tightly controlled by pro-resolving microglia to prevent collateral damage to surrounding neurons. However, during neuroinflammatory diseases, such as AD, this resolution of inflammation is dysregulated, resulting in chronic neuro-inflammation and subsequent neurotoxicity.

In AD, pattern recognition receptors on microglia trigger a pro-inflammatory immune response upon Aβ recognition (39). The inflammatory properties of Aβ are strengthened by promoting increased APP levels and elevated cleavage enzyme activity, creating more Aβ production (40). Additionally, microglia surrounding senile plaque become impaired in Aβ uptake and clearance, causing further accumulation of Aβ thereby inducing a prolonged inflammatory response with continuous secretion of pro-inflammatory mediators (41). The local immune response triggers the secretion of pro-inflammatory mediators such as TNF-α, IL-1β that subsequently activate astrocyte-induced proinflammatory responses. In turn, astrocytes amplify the microglia inflammatory responses by producing IL-1β and TNF-α upon activation (42). This, together with Aβ deposition and ROS formation, has considerable detrimental effects on the BBB, such as the loss of tight junctions, pericyte death, and a decrease in the coverage of the parenchymal basal membrane by astrocytic endfeet (43–46). In turn, this greatly abolishes BBB homeostasis and increases innate and adaptive immune cell trafficking toward the CNS, thereby contributing to excessive neuro-inflammation and cognitive impairment in AD (47–50). Creating insights into ways to counteract chronic neuroinflammatory events are of high importance to dampen disease progression.

## Sphingolipid Metabolism

The CNS has the second-highest abundancy of lipids to adipose tissue, with 50% of its dry weight comprising of lipids (51). They can be classified into 8 groups containing distinct classes and subclasses of molecules, performing key biological functions (52). Especially SLs gained interest in recent years because of their role as secondary messengers in health and disease. Not only are they ubiquitous components of the plasma membrane of eukaryotic cells and are essential for the development of the CNS, they are also known as bioactive lipids regulating cell survival, cellular stress and cell death (53–55).

**Figure 1:**
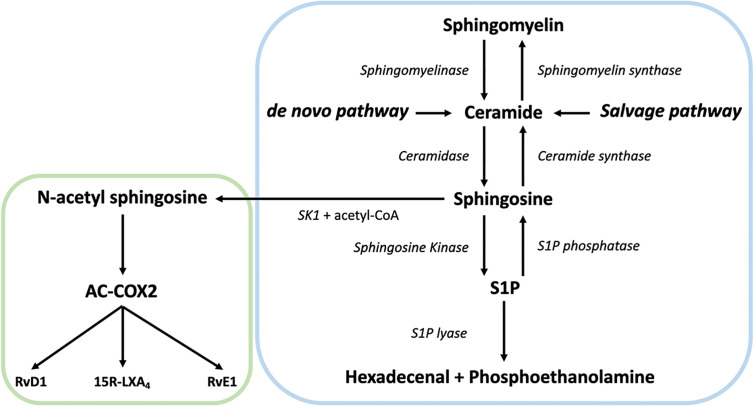
Overview of the sphingolipid (SL) rheostat model and the interplay with specialized pro-resolving mediator (SPM) metabolism. Ceramide can be synthesized by ceramide synthases *via* the *de novo* and the salvage pathway from sphingosine or by hydrolysis of sphingomyelin by sphingomyelinase. Once generated, ceramide can act as substrate for other sphingolipids such as sphingosine and sphingosine-1-phosphate (S1P) *via* sphingosine kinase (SK). S1P can be catabolized into hexadecenal + phospho-ethanolamine by the action of sphingosine 1-phosphate lyase. Alternatively, SK can generate N-acetyl sphingosine *via* acetyl-CoA and sphingosine, followed by the acetylation of COX-2. In turn, this activates COX-2 mediated 15-HETE, 18-HEPE and 17-HDHA production, which can be converted to SPMs like such as 15R-LXA_4_, RvE1, and RvD1, thereby providing a direct link between the SL and SPM pathways.

In the centre of SL metabolism are ceramides, that consist of a sphingosine backbone and a fatty acid residue. Ceramides can be synthesized *via* the *de novo* pathway, the sphingomyelinase pathway, or the salvage pathway (**Figure 1**). The *de novo* synthesis pathway starts with L-serine and palmitoyl-CoA condensation in the endoplasmic reticulum by serine palmitoyltransferase (SPT) to 3-ketosphinganine, that is directly reduced to sphinganine by 3-ketosphinganine reductase (3-KSR). Next, ceramide synthases (CerSs) add fatty acyl-CoAs of different chain lengths to sphinganine to form dihydroceramide. Finally, dihydroceramide desaturase converts dihydroceramide to ceramide. After ceramide synthesis, it can be further metabolized to form complex SLs, such as sphingomyelin and glycosphingolipids (56). These complex SLs create a potential ceramide source since they can be converted to ceramide again. For instance, the sphingomyelinase pathway generates ceramides *via* the hydrolysis of sphingomyelin. This is catalyzed by two sphingomyelinases, named neutral sphingomyelinase (nSMase) and acid sphingomyelinase (aSMase) (57, 58). Finally, ceramide can also be generated from sphingosine *via* the salvage pathway. While sphingosine can be reused to generate ceramide, it is also the precursor of sphingosine-1-phosphate (S1P) (59). The breakdown of S1P into non-sphingolipid molecules by S1P lyase is the only exit point of sphingolipid metabolism. Over the last decades, it became clear that SLs and their metabolites play an important role in several cellular processes and signaling events, including neuro-inflammation (60–62).

## Sphingolipids and Neuroinflammation

Ceramide and S1P are the main signaling molecules of the SL machinery that can activate a pro- or anti-inflammatory response. Activation of their modulators, such as SMase and sphingosine kinase (SK), are therefore important events during neuroinflammation. Originally it was thought that ceramide functions as a secondary messenger with two faces, where short-chain ceramides (acyl chain length C2-C8) show an anti-inflammatory effect while long-chain ceramides (acyl chain length C16-C24) initiate a pro-inflammatory response (63–66). However, synthetic short-chain ceramides were used to mimic the effects of long-chain ceramides resulting in contradictory results. For instance, the use of short-chain ceramides caused an anti-inflammatory effect in LPS stimulated rodent microglia, by competing with LPS for the binding to toll-like receptor-4 (TLR4). This resulted in the reduction of cytokines, chemokines, inducible NO synthase, cyclooxygenase-2 (COX-2, also known as prostaglandin G/H synthase 2) and ROS (63, 67, 68). In contrast, astrocytes and microglia produce long-chain ceramides upon TNF-α induced SMase activity. These ceramides activate pro-inflammatory transcription factor NF-κB, inducing expressions of pro-inflammatory cytokines such as IL-1*β*, IL-6, IL-8, NO, TNF-α, monocyte chemoattractant protein-1, pro-inflammatory enzyme cyclooxygenase-2 (COX-2), and lipoxygenases (LOXs) (64, 65, 69). These observations are supported by SMase knockdown experiments in rodent LPS-activated microglia, showing impaired NF-κB induced gene expression (66).

However, in the context of ceramide function in the brain, it has become clear that the amount of ceramide is important as well as the relative amount of the individual chain-lengths (70). The various ceramide species are generated by six individual CerSs, of which five are present in the brain. Each CerS prefers certain fatty acyl-CoA substrates, generating distinct ceramide species with unique *N*-linked fatty acids. The resulting ceramide species differ in their chain-length (C14-C26), localize to distinct cellular compartments, and in turn may mediate specific functions (71, 72). Therefore, contributing opposing functions to short- or long-chain ceramides is to simplified and the underlying regulatory process is far more complicated. This needs to be considered when investigating the role of ceramide in neuro-inflammation.

Besides ceramides, S1P plays an important role in the intracellular and extracellular signaling in the CNS. Various reports suggest that S1P is involved in migration, proliferation and changes in astrocyte and microglia morphology, suggesting its involvement in neuroinflammation (73). Upon activation, two distinct enzymes, SK1 or SK2, phosphorylate sphingosine to form S1P. Although S1P has several intracellular targets, S1P is predominantly transported to the outside the cell, where it acts in a paracrine or autocrine manner on five different S1P receptors (S1PR1-5), which are G protein-coupled receptors (74). S1PR1-3 are ubiquitously expressed while S1PR4 is mainly expressed by leukocytes and S1PR5 by oligodendrocytes and brain endothelial cells (75, 76).

Upon LPS induced activation, SK1 shuttles to the plasma membrane where it converts sphingosine to S1P. Subsequently, S1P binds to S1PRs, which induces proliferation and synthesis of pro-inflammatory cytokines, such as TNF-α, IL-1β, and IL-17, and neurotoxic molecules like ROS and NO (77). Additionally, the accumulated extracellular S1P activates microglia and further enhances the inflammatory response (78). Also, S1PR, and SK1 knockdown or the addition of S1PR antagonists reduce pro-inflammatory responses (79, 80). At the level of the BBB, different S1PRs seem to be involved in the remodeling of its integrity. Endothelial cells express three types of S1PRs, S1PR1 activation restricts leukocyte infiltration to the CNS while S1PR2, 3 and 5 regulate vascular permeability by enhancing pro-inflammatory expression. Astrocyte-endothelial cell communication *via* S1P and/or ceramide may, therefore, be important in maintaining BBB homeostasis as it can promote or decrease its integrity (81–85). This shows that directing specific S1PR activation may influence inflammatory responses in the CNS.

## Sphingomyelinase and Ceramide During Neuroinflammation in Alzheimer’s Disease

In AD, many of the SLs and their metabolites are altered. For example, increased sphingomyelin levels are observed in brain tissue of AD patients, which is associated with the severity of AD pathology (86). However, SMase levels and activity are also increased due to the presence of Aβ and, therefore, could result in increased sphingomyelin hydrolysis (87). Moreover, elevated aSMase significantly correlated with the levels of Aβ and hyperphosphorylated tau protein (88). The enhanced SMase levels in AD are possibly involved in pro-inflammatory processes in the brain. The inhibition of nSMase, not aSMase, in Aβ activated human astrocytes suppresses the production of NF-κB and pro-inflammatory cytokines, such as TNF-α, IL-1*β* and IL-6 (89). Additionally, antisense knockdown of nSMase lowered inducible NOS *in vivo* and protected neurons in the mouse cortex from fibrillar Aβ toxicity. This indicates that nSMase has a role in the pro-inflammatory activation of astrocytes through a nSMase/ceramide signaling pathway. In addition, exosomes secreted by activated astrocytes induced apoptosis in surrounding astrocytes by transporting long-chain ceramide C18 (90). This toxic effect was attenuated upon nSMase inhibition, suggesting nSMase activation results in a neurotoxic ceramide secretion *via* exosomes. Furthermore, the increased activity of the sphingomyelin pathway is a large source of ceramide observed in AD (87).

Involvement of the *de novo* ceramide synthesis pathway is also reported in AD. SPT is the first enzyme in the *de novo* synthesis of ceramide, and elevated SPT long-chain 1 and SPT long-chain 2 levels are observed in AD (91). Inhibition of SPT directly lowers ceramide synthesis and results in decreased Aβ production, which supports the findings that ceramide metabolism is involved in amyloidopathy (92). For example, ARN14494, which inhibits SPT activity, prevents the synthesis of long-chain ceramides and dihydroceramide in a cortical astrocyte-neuron co-culture. Blockade of SPT activity also prevents the synthesis of pro-inflammatory mediators, such as IL-1*β*, TNF-α, iNOS, and COX-2 by astrocytes. Additionally, inhibition of SPT possibly prevents caspase-3 neurotoxicity, *via* the reduced expression of astrocyte secreted pro-inflammatory factors (93). This suggests that ceramide induces pro-inflammatory responses through astrocytes, which may promote neurotoxicity. The exact mechanism by which ceramide activates the pro-inflammatory astrocyte response remains to be established.

Indeed, increased long-chain ceramide levels have been found in AD affected brains, senile plaques, cerebral spinal fluid (CSF) and serum of AD patients (94–98). Interestingly, ceramides enhance APP metabolism toward Aβ by stabilizing β-secretase, creating a vicious cycle. This results in increased ceramide levels in neurons possibly leading to cell death. These observations indicate that interfering in ceramide synthesis possibly reduces Aβ pathology and neuronal cell death in AD (99–101). Taken together, the *de novo* and the sphingomyelinase ceramide synthesis pathways show to increase the expression of pro-inflammatory cytokines and chemokines in AD. Ceramide metabolism might therefore be an interesting therapeutic target to prevent and resolve neuroinflammation during AD.

## Sphingosine-1-Phosphate During Neuroinflammation in Alzheimer’s Disease

The function of S1P in AD affected brains remains controversial. Analysis of *post-mortem* brain tissue of AD patients showed a reduced level of S1P, which correlated with the levels of hyperphosphorylated tau and Aβ (88). The reduction might be caused by decreased SK1 and increased S1P-lyase activity due to Aβ, which supports the idea that S1P is a pro-survival and proliferative signal (102, 103). On the other hand, however, prolonged exposure of hippocampal neurons to S1P resulted in apoptosis (104). Moreover, depletion of S1P lyase *in vivo*, causing an increase in S1P levels, augments tau phosphorylation in neurons (105). A study focusing on the development of AD showed elevated S1P levels in mild cognitive impairment patients while eventually in AD patients, S1P levels declined (106). Interestingly, another study investigated whether sphingolipid levels are altered as a function of age and *APOE* genotype (107). The authors observed an age-dependent decline in S1P levels specifically in females. Moreover, the APOE genotype was not found to have a significant influence on the SL levels. These findings suggest that age is an important factor regarding SL metabolism, where increased S1P levels might play a role in the early development of AD and, as observed in post-mortem AD brains, these levels decrease over time.

Zhong and colleagues proposed a mechanism that displays how S1P is involved in pro-inflammatory activation of microglia in AD (108). They showed that Aβ activates spinster homolog 2 (Spns2), which transports S1P out of the cell, resulting in the subsequent binding of S1P to S1PR1. S1P binding to S1P1R induced the pro-inflammatory cytokine secretion of microglia *via* a NF-κB dependent mechanism. These experiments were conducted *in vitro* and *in vivo*, using primary cultured microglia, mouse models, Spns2 knockout mice as well as an S1PR inhibitor Fingolimod (FTY720). Spns2 knockout mice show reduced inflammatory microglia phenotypes, suggesting that S1P transport is important for the activation of microglia and provides evidence that S1P contributes to Aβ-induced NF-κB signaling and cognitive decline. Another study used LPS activated microglia and astrocytes to study S1PR1 dependent pro-inflammatory chemokine release. Here, the inhibition of S1PR1 *via* FTY720 attenuates pro-inflammatory chemokine release in both astrocytes and microglia (109). Interestingly, LPS binds to TLR4 to activate pro-inflammatory responses, which suggests that TLR4 may mediate pro-inflammatory cytokine/chemokine secretion *via* S1PR1 activation.

In astrocytes, TLR4 seems to activate SKs resulting in chemokine expression (110). This could also be true for Aβ induced NF-κB secretion *via* S1PR activation since Aβ binds to TLR4, TLR2 and CD14 for the pro-inflammatory activation of microglia (39, 111, 112). Indeed, in case Aβ would mediate pro-inflammatory responses *via* other mechanisms, a stronger pro-inflammatory effect could be expected in FTY720 inhibited microglia and astrocytes (108). In conclusion, S1P signaling through S1PR1 seems to play a pivotal role in the pro-inflammatory responses by microglia and astrocytes. The onset of Aβ induced neuroinflammation through TLR/SK/S1P/Spns2/S1PR1/NF-κB signaling possibly takes place in the early stages of AD, as S1P levels are higher in mild cognitive impairment patients before the official onset of AD. However, the exact mechanisms behind the onset of neuro-inflammation in AD by S1P remains to be established.

## Specialized Pro-Resolving Mediators and the Resolution of Neuroinflammation

Resolution of inflammation is crucial to regain tissue homeostasis. When resolution fails, chronic inflammation occurs, causing excessive release of pro-inflammatory cytokines and mediators, potentially leading to ongoing neuroinflammation and neurodegeneration, as seen in AD (113). Under healthy conditions, the resolution of inflammation is facilitated by SPMs that are derived from polyunsaturated fatty acids (PUFAs). These include ω-3 fatty acids, like α-linolenic (ALA) acid, docosahexaenoic acid (DHA), and eicosapentaenoic acid (EPA) as well as ω-6 fatty acids, such as linolenic acid (LA) and arachidonic acid (AA). The PUFAs are predominantly metabolized by lipoxygenases (LOX) and, to a lesser extent by (acetylated) cyclooxygenases (COX) to generate SPMs, such as lipoxins, E-series resolvins, D-series resolvins, protectins and maresins (114). In general, SPMs are potent resolution agonists that extinguish the eicosanoid-induced inflammation by activating local resolution programs, eventually leading to tissue recovery (115, 116).

During an acute inflammatory event, the vasculature as well as local macrophages/microglia are activated, resulting in the production of pro-inflammatory cytokines as well as the activation of lipid mediator producing enzymes, such as COX and LOX. In general, COX activity supports the secretion of prostaglandins, like PGE_2_ and PGI_2,_ leading to the migration of leukocytes, such as neutrophils to the site of inflammation. In this initial pro-inflammatory response, leukotriene B4 (LTB_4_) is produced by innate immune cells, also attracting leukocytes toward the site of inflammation. This pro-inflammatory lipid mediator response is changed to a pro-resolving response in a process called lipid mediator class switching (117). In particular, this consists of the change of AA metabolism from pro-inflammatory LTB_4_ to a pro-resolving lipoxin A_4_ (LXA_4_) lipid mediator production in response to inflammation (e.g., eosinophils) or due to a phenotype switch (e.g., macrophages). Four LXA_4_-biosynthesis pathways that are involved in class switching are currently known. First, protein kinase A gets activated by PGE_2_ resulting in the phosphorylation of 5-LOX. This increases LXA_4_ synthesis and inhibits LTB_4_ (118). Secondly, neutrophils induce 15-LOX-mediated LXA_4_ synthesis and downregulate 5-LOX mediated LTB_4_ synthesis, induced by PGE_2_ (115). Thirdly, endotoxin or extracellular ATP can induce hydrolytic release of the esterified 15-HETE and synthesize LXA_4_ *via* 5-LOX pathway (119). Finally, the activity of 12/15-LOX can catalyze LTA_4_ conversion to LXA_4_ (120). Overall, increasing LXA_4_ synthesis contributes to the decreased leukocyte migration toward the site of inflammation and is, therefore, the first step in the resolution response.

The E and D series resolvins, protectins, and maresins, derived from ω-3 fatty acids, are metabolized by LOX and/or CYP450 (115, 121). These SPMs are locally secreted to stimulate macrophage/microglia phenotype switching toward a pro-resolving phenotype. In turn, this promotes efferocytosis for the clearance of debris and downregulates the activity of the adaptive immune system to facilitate the return to tissue homeostasis (122, 123). Therefore, anti-inflammation and pro-resolution are not equivalent. The SPMs that actively promote resolution are fundamentally different from the antagonists that limit the duration and magnitude of the inflammatory response at both the molecular and cellular levels (124).

## Specialized Pro-Resolving Mediators in AD

An important process in the return to tissue homeostasis after the onset of acute inflammation is inflammation resolution *via* SPMs. When resolution fails, acute inflammation will acquire a chronic phenotype, resulting in severe tissue damage. Chronic inflammation in AD is possibly caused by alterations in the SPM production machinery (125). Of note, it was shown that APOE4 may mechanistically impact the neuropathogenesis of AD by decreasing DHA transport into the brain, which in turn, may lead to lower SPM levels in patients (126). Understanding the mechanisms behind this resolution failure can therefore be of clinical value for the treatment of AD.

Currently, only a few studies have addressed the potential effects of SPMs in AD. For example, lower levels of LXA_4_ are found in *post-mortem* hippocampal tissue as well as CSF compared to controls (125). Additionally, CSF levels of LXA_4_ and RvD1 are correlated with the mini-mental state examination scores, suggesting that the impaired resolution of neuroinflammation is involved in the cognitive decline in AD (127). Additionally, higher levels of 15-LOX-2, 15-LOX-1 and 5-LOX enzymes are observed in AD hippocampus. However, these enzymes are also known to mediate the production of pro-inflammatory lipid mediators and depend on class switching to generate SPMs (117). Therefore, it is possible that the increase of 15-LOX and 5-LOX together with the lack of lipid class switching facilitates the ongoing pro-inflammatory response (125, 128, 129). This also suggests that AD progression might be reduced when altered local SPM levels are restored. Indeed, treatment with aspirin-triggered LXA_4_ was shown to ameliorate Aβ and tau pathology *in vivo* (130). In addition, enhancing LXA_4_ signaling by using aspirin leads to reduced pro-inflammatory cytokine and chemokine levels, while anti-inflammatory IL-10 levels are elevated, leading to more pro-resolving microglia phenotypes, Aβ phagocytosis and improved cognition (127). Similar to LXA_4_, RvD1 induces an pro-resolving microglia phenotype and enhances microglia-mediated Aβ phagocytosis (131). Besides LXA_4_, maresin-1 is also decreased in *post-mortem* hippocampal tissue and CSF of AD patients compared to controls (125). Importantly, *in vitro* experiments with the CHME3 microglial cell line revealed enhanced Aβ phagocytosis and attenuated microglia activation when incubated with maresin-1 (132). Together, these findings suggest that administering disease-affected SPMs or activating local SPM biosynthesis during AD could be an interesting therapeutic approach to resolve chronic inflammation and thereby prevent neurodegeneration.

## Sphingolipid Mediated Resolution of Neuroinflammation *via* SPMs

So far, only few studies have reported on the interplay of the SL and SPM machinery and the role thereof in AD is now emerging. Interestingly, Young Lee and colleagues demonstrated a direct anti-inflammatory correlation between the SL machinery and SPMs in AD (133, 134). Neuronal SK1 appears to generate N-acetyl sphingosine *via* acetyl-CoA and sphingosine, followed by the acetylation of serine residue 565 of COX-2 by N-acetyl sphingosine. This activates COX-2 mediated 15-HETE, 18-HEPE, and 17-HDHA production, which can be converted to SPMs, such as 15R-LXA_4_, RvE1, and RvD1. In APP/PS1 mice, SK1 is severely decreased in neurons but not in microglia, causing a decrease in N-acetyl sphingosine and therefore a decline in SPM production and secretion by neurons. The increased or decreased SK1 levels result in altered SPM levels as well as phagocytosis of Aβ by microglia in APP/PS1 mice, respectively (133, 134). Of note, SK1 levels appear to be reduced in *post-mortem* AD affected brains (103, 133). This suggests that neuronal SK1 fulfils an anti-inflammatory role during neuroinflammation in AD. Additionally, N-acetyl sphingosine is decreased in microglia, caused by deficient acetyl-CoA, reducing acetylated COX-2 and SPM secretion by Aβ activated microglia from C57BL/6 mice. Treating 5xFAD and APP/PS1 mice with N-acetyl sphingosine increased COX-2 acetylation and subsequent SPM biosynthesis in microglia (134). This facilitates the resolution of neuroinflammation and enhances the phagocytosis of Aβ by microglia. Overall, these findings indicate that the sphingolipid machinery has an immune regulatory function by activating SPM biosynthesis in the CNS *via* COX-2 acetylation (**Figures 1** and **3**). Moreover, the immune regulation *via* sphingolipids seems to be dysregulated in AD, providing a new framework to reinstate the resolution of neuroinflammation in AD.

## Sphingolipid and SPM Based Therapeutic Approaches for AD

Although the fundamental knowledge about SLs and SPM metabolism in the CNS during healthy and pathological situations remains to be fully elucidated, it has been demonstrated that changes in their metabolic pathways occur during AD pathogenesis as described above. In turn, this paves the way to include their receptors, metabolites, and involved machinery as possible therapeutic targets to limit the progression of AD. Various strategies targeting these pathways will be discussed below (see **Table 1** for complete overview).

**Table 1:** Sphingolipid and SPM based therapeutic approaches for AD.

Compound	Target	In vivo/*in vitro*	Cell type/animal model	Concentration	Reference
Fingolimod	S1PR1, 3, 4, 5	*In vitro*	Mouse neuronal cultures	1–100 pM	(135)
		*In vivo*	5XFAD mouse model	0.03–5 mg/kg/day	(136, 137)
		*In vivo*	APP/PS1 mouse model	0.3 mg/kg/day	(138)
ARN14494	SPT	*In vitro*	Aβ induced cortical astrocyte-neuron co-cultures	1, 5, 10 µM	(93)
L-cycloserine	SPT	*In vitro*	cortical neurons and astrocytes	2 mM	(92)
Myriocin	SPT	*In vitro*	human oligodendroglioma cell line	5 µM	(139)
PDDC	nSMase2	*In vivo*	5XFAD+vehicle mouse models	10 mg/kg/day	(140)
GW4869	nSMase2	*In vitro*	hippocampal neuronal cultures	150 µM	(141)
Cambinol	nSMase2	*In vitro*	hippocampal neuronal cultures	0.1–30 µM	(141)
Aspirin	COX2	*In vitro*	Mice microglia	10, 100, 1000 nmol/liter	(127)
		*In vivo*	Tg2576 mice	15 µM/kg 2x per day	(127)
Defensamide	SK1	*In vitro*	primary cultured human keratinocytes	100 µM	(142)
AE1-329	EP4	*In vitro*	primary cultured mouse microglia	100 µM	(143)

## Potential S1P Metabolism-Related Therapeutic Targets and Therapies

S1P signaling through S1PR1 demonstrated to be a possible initiator for pro-inflammatory immune responses of microglia (108). Inhibition of S1PR1 signaling could, therefore, be an interesting approach to attenuate neuroinflammation in AD. Fingolimod (FTY720) is a functional antagonist that promotes initial activation followed by sustained internalization and desensitization of several S1PRs in lymphocytes, except S1PR2 (144). Fingolimod is approved by the European Medicines Agency (EMA) as a treatment for relapsing-remitting multiple sclerosis (MS) and has the potential to target major processes in AD pathogenesis as well, including Aβ toxicity and production, neuroinflammation and neuronal loss. *In vitro* experiments demonstrated that Fingolimod ameliorates Aβ toxicity in neuronal cultures *via* increased concentrations of brain-derived neurotrophic factor (135, 145). *In vivo* models, using the 5XFAD transgenic AD mouse model, displayed decreased signs of neuroinflammation and cognitive improvement when given a low dose (0.03 mg/kg/day) (136, 137). Other experiments demonstrated that Fingolimod attenuates pro-inflammatory chemokine release in both astrocytes and microglia (108, 109). Furthermore, Aβ load is decreased in APP/PS1 mice by the inhibition of β-secretase when treated with Fingolimod, possibly by modulating the transport of Aβ through the BBB (138). Taken together, this shows that Fingolimod is a promising new therapeutic approach for AD. Moreover, other neurodegenerative or neuro-inflammatory diseases such as Parkinson’s disease, Huntington’s disease, and epilepsy also explore the use of Fingolimod as possible treatment because of its diverse anti-inflammatory and neuroprotective effects (146). However, although different animal models show promising results upon treatment with Fingolimod, further experiments, as well as clinical studies, should elucidate if patients indeed benefit from Fingolimod as medication.

**Figure 2:**
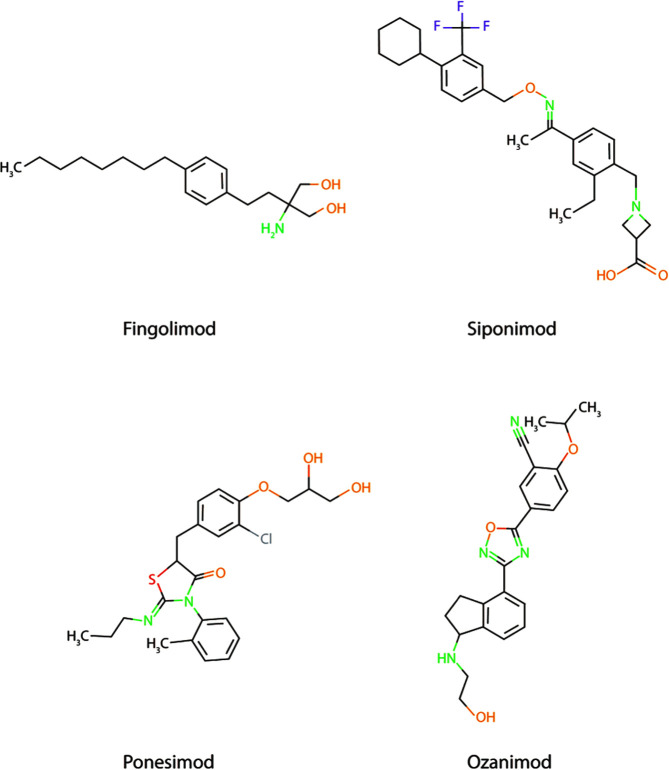
Chemical structure of Fingolimod together with three other more selective S1PR inhibitors; Siponimod, Ponesimod, and Ozanimod.

The promising preclinical results of S1PR inhibitor Fingolimod also creates possibilities for the use of other more selective S1PR inhibitors, such as Ponesimod (acts *via* S1PR1), Siponimod (acts *via* S1PR1 and S1PR5), and Ozanimod (acts *via* S1PR1 and S1PR5) in AD (**Figure 2**), especially since Fingolimod targets all S1P receptors (except S1PR2), creating potential harmful side effects (147, 148). Currently, the use of these S1PR inhibitors are focused on therapy development for MS and no scientific literature is describing their use in AD models. Another strategy to interfere in the S1P signaling could be *via* the inhibition of the S1P transporter Spns2. As described earlier, Spns2 knockout mice display reduced inflammatory microglia phenotypes and Spns2 is possibly involved in the Aβ42-induced NF-κB signaling and cognitive decline. Additionally, Spns2 forms a complex with major facilitator superfamily domain-containing 2a (Mfsd2a) to optimize S1P transport and shows involvement in maintaining BBB integrity by adjusting S1P concentrations (149). This indicates that S1P transport could potentially be inhibited *via* either Spns2 or Mfsd2a. Unfortunately, no inhibitors are currently available for both Spns2 and Mfsd2a.

## Ceramide Synthesis Inhibition as a Therapeutic Target in AD

With enhanced levels of long-chain ceramides found in AD, inhibition of ceramide metabolism could be an interesting therapeutic approach. For instance, targeting the *de novo* ceramide synthesis by inhibiting SPT has already been investigated by using SPT inhibitors such as myriocin, ARN14494 and L-cycloserine. *In vitro* experiments with myriocin indicated that it inhibits ceramide synthesis *via* SPT in MS and its mouse model experimental autoimmune encephalomyelitis (139). However, myriocin has not been extensively tested for efficacy in AD models. In AD *in vitro* models, ARN14494 and L-cycloserine are capable of inhibiting SPT, resulting in decreased ceramide and pro-inflammatory cytokine levels (92, 93). While these initial *in vitro* results are promising, studies exploring the effect of SPT inhibition *in vivo* is needed to confirm whether these inhibitors have indeed anti-inflammatory and neuroprotective effects.

Targeting the sphingomyelinase pathway might be another approach to decrease ceramide levels. For instance, the knockdown of nSMase in Aβ activated astrocytes decreased their pro-inflammatory cytokine and chemokine secretion (89). GW4869 and Cambinol are proven inhibitors of nSMase and show neuroprotective and anti-neuroinflammatory properties (90, 141). However, these inhibitors have an unfavorable IC_50_ of >1µM. Additionally, GW4869 is insoluble and has a high molecular weight, creating difficulties for the conduction of (pre)clinical studies (141, 150). Recently a new nSMase2 inhibitor was identified, phenyl (R)-(1-(3- (3,4-dimethoxyphenyl)-2,6-dimethylimidazo[1,2-b]pyridazin-8-yl)- pyrrolidin-3-yl)carbamate 1 (PDDC). This inhibitor showed in the 5XFAD+vehicle AD mouse model that it can penetrate the BBB, inhibit exosome release and neuroinflammation (140, 151). The first results with PDDC as nSMase2 inhibitor seem promising but, being a new compound, additional research is warranted.

Overall, the inhibition of ceramide synthesis pathways shows potential to function as a therapeutic approach for AD. A reduction of pro-inflammatory cytokines and chemokines is observed upon the use of ARN14494 and L-cycloserine to inhibit *de novo* synthesis *in vitro*. Additionally, PDDC already showed inhibitory effects on SMase ceramide synthesis pathways, decreasing neuroinflammation *in vivo.*

## Boosting the Resolution of Neuroinflammation as a Novel Treatment Modality for AD

The exploitation of SPMs to resolve neuroinflammation in AD is still in its infancy. Several papers demonstrate that aspirin can acetylate COX-2, resulting in the blocking of prostaglandin biosynthesis and activation of SPM biosynthesis. For example, aspirin-triggered LXA_4_ production reduced NF-κB activation and pro-inflammatory cytokine and chemokine secretion in aspirin treated microglia. It also increased Aβ phagocytosis by microglia and improved cognitive function in Tg2576 mice (127). Aspirin is currently the only known therapeutic that inhibits the pro-inflammatory response and activates the anti-inflammatory response of COX-2. However, a clinical human trial showed no evidence that aspirin reduces the risk of AD (152).

Other therapeutic approaches could consist of SK1 activation *via* (S)-Methyl 2-(hexanamide)-3-(4-hydroxyphenyl) propanoate (MHP), also known as Defensamide (142). Young Lee and colleagues demonstrated how SK1 has a pro-resolving effect on neuroinflammation *via* N-acetyl sphingosine generation followed by COX-2 acetylation, resulting in SPM biosynthesis (133, 134). It can therefore be hypothesized that activation of SK1 by Defensamide might be a novel SPM promoting therapeutic approach. Currently, Defensamide was shown to activate SK1 in human keratinocytes (an epidermal cell line), however, it is not known if this activation also increases N-acetyl sphingosine generation and no publications discuss its effect in an AD experimental setup (142). The effect of Defensamide remains, therefore, to be established.

An important event in the resolution of neuroinflammation is lipid mediator class-switching. This can for example be induced by the activation of E-prostanoid (EP)4 receptor by PGE_2_. In turn, this enhances LOX-15 production that induces LXA_4_ biosynthesis (153). This indicates that activation of EP4 during neuroinflammation in AD could represent a new therapeutic approach. Indeed, AD *in vitro* microglial experiments showed that EP4 receptor activation by EP4 receptor agonist AE1–329 attenuates Aβ induced ROS, pro-inflammatory cytokine and chemokine expression. Additionally, EP4 receptor expression levels seem to be reduced in human *post-mortem* AD brain (143). This indicates that activating the EP4 receptor *via* AE1-329 might be a possible new therapeutic route for the resolution of neuroinflammation during AD, but the lowered expression of EP4 may attenuate its effects. Currently, only one paper discusses the effect of AE1-329 in a mouse model of cerebral ischemia, confirming that AE1-329 does enter the brain and therefore could be implemented in AD mouse model studies (154). Overall, research into new therapeutics for the targeting of SPM metabolism in AD is still at the beginning, but some publications show that modulation of the SPM metabolism can be applied as a potential novel treatment strategy.

## Discussion and Future Directions

**Figure 3:**
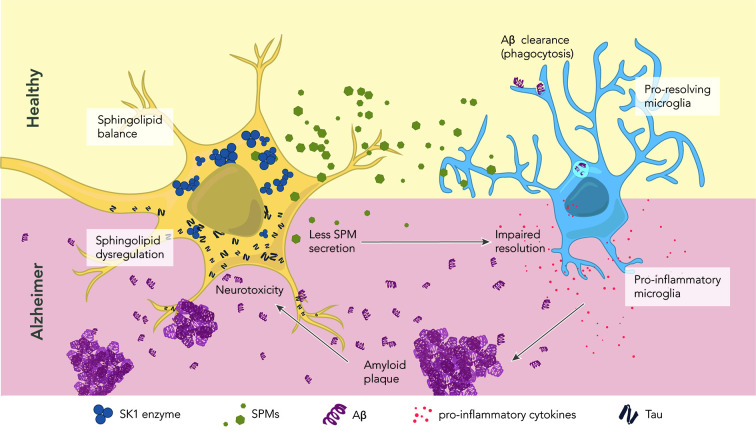
The role of sphingolipids and specialized pro-resolving mediators in health and disease. In healthy conditions, neurons maintain a proper balance of sphingolipids. The abundant SK1 enzyme deviates the sphingolipid pathway toward SPM production and secretion. Secreted SPMs reach perineuronal microglia, promoting their pro-resolving phenotype. Pro-resolving microglia maintain a healthy microenvironment by clearing amyloid beta through phagocytosis. In AD, there is a dysregulation of sphingolipids and SPMs, which correlates with the levels of hyperphosphorylated tau and Aβ. Reduced levels of the enzyme SK1 result in less SPM production and secretion. Microglia become pro-inflammatory, and start secreting pro-inflammatory cytokines. Aβ is no longer cleared, leading to the formation of extracellular amyloid plaques. These plaques further contribute to neurotoxicity.

Our understanding of neuroinflammation in AD has tremendously increased over the last decade. With this, it became clear that both SL and SPM metabolism are major players in the onset and resolution of excessive neuroinflammation during AD. Increased ceramide and SMase levels are found in AD brain and showed to be part of the signaling cascades for pro-inflammatory responses (87, 95). S1P signaling in AD remains controversial, as levels are increased in mild cognitive impairment patients but are attenuated in cases with more advanced AD (104). The exact mechanisms underlying SL metabolism alterations in AD patients is largely unknown. Additionally, several SPMs are reduced in AD patient tissues and body fluids, suggesting potential defects in resolution pathways, but how this decrease in SPM levels is mediated remains to be established. Interestingly, it was suggested that SK1 can generate N-acetyl sphingosine that acetylates COX-2, resulting in activation of the SPM production (133, 134). This suggests that SL metabolism is involved in the resolution of neuroinflammation *via* SPM biosynthesis, thereby providing a direct link between these bioactive lipid pathways (**Figures 1** and **3**). In short, although increased understanding of SL and SPM metabolism in AD is gained over the years, extended research should be conducted to further understand its involvement in AD pathology. This includes getting a better understanding of the underlying mechanisms that are involved in lowering SK1 and subsequent SPM levels, as well as increased ceramide levels.

Although the development of SL and SPM therapeutics is still in its infancy, several potential compounds show beneficial effects on reducing neuroinflammation, increasing Aβ phagocytosis, and decreasing the levels of phosphorylated tau (90, 92, 130, 135, 139–141, 145, 151). Fingolimod is one of those prime candidates, demonstrating decreased neuroinflammation in both *in vitro* and *in vivo* models of AD. Additionally, Fingolimod is already approved by the EMA as therapy for MS. This makes Fingolimod one of the most promising therapeutic compounds to reduce the pro-inflammatory response *via* S1PRs in AD. Therefore, additional research with AD models should be conducted, focusing on the inhibition of neuroinflammation using S1P receptor specific therapeutic compounds like Ponesimod, Siponimod, and Ozanimod.

Therapeutics that focus on the downregulation of ceramide syntheses, such as PDDC, ARN14494, and L-cycloserine, are possibly effective to fight progression of AD pathology, as ceramide levels appear to be increased throughout AD progression. However, Fingolimod and Defensamide are possibly most effective during the early stages of AD. For instance, SK1 levels are decreased in *post-mortem* brain tissue of AD patients. In addition, mild cognitive impairment patients show high S1P levels, but their levels decrease during the progression of AD. Therefore, the effects of possible treatments should be studied throughout AD progression to determine the most effective treatment window.

In conclusion, SL and SPM metabolism are essential players in the onset and resolution of neuroinflammation in AD (**Figure 3**). Increasing our knowledge about alterations in their metabolism and signaling, and more importantly about the interplay between SLs and SPMs metabolism will provide new perspectives for the development of innovative therapies for AD based on resolution pharmacology. It is therefore of major importance to gain more insight in the coming years into the underlying mechanism of action by which SLs and SPM signaling and metabolism act during tissue homeostasis and neuroinflammation in AD.

## Author Contributions

This manuscript was written by NW, KM, and GK. SRL provided the illustration. HV and SRL contributed in revising the manuscript. All authors contributed to the article and approved the submitted version.

## Funding

This work was supported by a grant from IMI (807015 to NW), a grant from the Dutch MS Research Foundation (20-1087 MS to SRL), as well as a grant from the Dutch Research Council (NWO Vidi grant 91719305 to GK).

## Conflict of Interest

The authors declare that the research was conducted in the absence of any commercial or financial relationships that could be construed as a potential conflict of interest.
